# Improving bedside dispensing services through early medication discharge planning: a pre-post intervention study

**DOI:** 10.1186/s40545-022-00405-3

**Published:** 2022-01-24

**Authors:** Ai Ling Oh, Yi Jing Tan, Wan Choon Chong, Irene Yee Yew Chieng, Jaime Yoke May Chan, Boon Phiaw Kho, Mei Ing Theng, Crystal Sing Yee Tan

**Affiliations:** Department of Pharmacy, Sarawak General Hospital, Ministry of Health Malaysia, Jalan Tun Ahmad Zaidi Adruce, 93586 Kuching, Sarawak Malaysia

**Keywords:** Bedside dispensing, Medication discharge planning, Medication errors, Medication reconciliation, Transition of care

## Abstract

**Background:**

Delays in producing discharge prescriptions have hindered the provision of bedside dispensing services (BEDISC) that enable medication reconciliation and pharmaceutical intervention, which is an important element in transitional care medication safety. We aimed to assess the impact of early medication discharge planning on the delivery of BEDISC in terms of the rate of bedside dispensing, medication errors, and cost-saving from medication reconciliation by reusing patient’s own medicines (POMs).

**Methods:**

A pre–post intervention study was conducted at medical wards in a public tertiary hospital. During the intervention phase, a structured bedside dispensing process was delineated and conveyed to the doctors, nurses, and pharmacists. Regular verbal reminders were given to the doctors to prioritize discharge patients by producing the prescriptions once discharge decisions had been made and nurses to hand the prescriptions to ward pharmacists and not patients. Throughout the study, ward pharmacists were involved in medication reconciliation via screening of discharge prescriptions and reusing POMs, performed pharmaceutical interventions for any medication errors detected, and provided bedside dispensing with discharge counseling. Comparisons were made between bedside versus counter-dispensing at pre–post intervention phases using the chi-square test.

**Results:**

A total of 1097 and 817 discharge prescriptions were dispensed in the pre-intervention and post-intervention phases, respectively. The bedside dispensing rate increased by 13.5% following remedial actions (*p* < 0.001). The number of prescriptions intervened due to detection of medication errors increased by 13.4% for bedside dispensing (*p* < 0.001) versus 4.7% for counter-dispensing (*p* = 0.002), post-intervention. Most medication errors fell under the category of inappropriate drug (44.5%), followed by inappropriate dose (12.8%). Reusing POMs resulted in cost-saving of MYR6,851.66 at pre-intervention and MYR7,032.98 at the post-intervention phase. Overall, the cost-saving from reusing POMs in both intervention phases was 52.7% (MYR13,884.64 out of the total MYR26.367.47), with the majority contributed by respiratory medications (40.2%) followed by cardiovascular (18%) and vitamins/minerals (17.5%).

**Conclusion:**

Pharmacist-coordinated early medication discharge planning has improved the delivery of bedside dispensing services, enhanced medication safety, and reduced medication costs.

## Background

Transitions of care between hospital and primary care settings have been recognized as high-risk scenarios that may jeopardize patient safety [1]. Studies showed that there were approximately 14–15% unintentional medication errors occurred post-discharge [2, 3]. This had led to adverse drug events, detection of medication continuity error post-discharge, and rehospitalization [2, 4, 5].

In the aspect of medication safety during transitions of care, reconciliation of medication has been recognized as one of the effective strategies to reduce medication errors, adverse drug events and prevent hospital admissions [6, 7]. This can be done either upon hospital admission, during internal ward transfer, or before discharge [8]. Medication reconciliation involves identifying current medications, comparing medications in the discharge prescriptions with current medications and pre-admission medications, making clinical decisions and therapeutic recommendations based on the patient’s clinical condition to formulate a complete and optimized drugs regimen [1]. Medication reconciliation involving past medications sorting and reusing patient’s own medications (POMs) helps to prevent confusion between past and present medications and reduce drugs wastage [9, 10]. Using POMs instead of routine dispensing has resulted in substantial cost savings, with up to 74% reduction in hospital drugs expenditure recorded in a Canadian hospital [11]. Medications cost savings have outweighed the labor costs of performing medication reconciliation and it has proven to be a cost-effective strategy by optimizing pharmacotherapy and preventing medication errors [12, 13].

Another important strategy to ensure medication safety is bedside dispensing and counseling services provided by pharmacists upon discharge. It was shown that increased medication knowledge and compliance, especially to those with multiple medications via discharge counseling provided by the pharmacist was able to reduce hospital readmissions and emergency department visits [14, 15]. This portrayed that pharmacists could serve as the last line of defense in transitional care medication safety. Most of the time, patients have been counseled on some medications, and devices during ward stay but no reinforcement was given upon discharge. This could have led to non-compliance due to changes in medication regimen upon discharge without patient knowledge and inadequate patient education [16, 17].

## Rationale of study

In our hospital setting, there were delays in producing discharge prescriptions due to a lack of prioritization to handle discharge patients. Therefore, patients or caregivers have to obtain the discharge medications at pharmacy counters as ward pharmacists were unable to perform bedside dispensing due to the late discharges. Medication reconciliation upon discharge, which included medication screening for potential medication errors and re-use of POMs, was also unable to be implemented as actively as upon admission, mainly due to the delayed discharge process.

Therefore, a structured bedside dispensing process with early medication discharge planning is imperative to ensure this service can be carried out smoothly and effectively. With the implementation of a structured process via a multidisciplinary approach, we seek to increase bedside dispensing rate, reduce medication errors, increase cost savings by re-use of POMs, and enhance patient’s medications understanding and adherence through proper discharge counseling provided.

## Methods

### Design, setting and population

A pre–post intervention study was conducted over 9 months at a tertiary referral center in Borneo Island, Malaysia consisting of four general medical wards covered by ward pharmacists in each ward. A total of seven ward pharmacists with at least 3 years of working experience were involved in data collection throughout the study period from pre- to post-interventional phase. All ward pharmacists had been briefed and trained regarding the clinical tasks, duties, and responsibilities to ensure their competencies in carrying out the daily routine.

All adult patients being discharged from general medical wards during office hours (8 am–5 pm) from Monday to Friday, excluding public holidays, were included in the study, except those who refused or were unfit (e.g., unconscious and mentally challenged) and without caregiver around to be provided with bedside dispensing service.

This research was registered with the National Medical Research Register bearing the registration number NMRR-18-3303-45025, ethical approval was exempted by the Medical Research and Ethics Committee (MREC), Ministry of Health Malaysia.

### Data collection

This study was divided into three phases. The pre-intervention phase involved data collection over 3 months from 1 Oct till 31 Dec 2018. This was followed by a 3-month intervention phase from 1 Jan till 31 Mar 2019, with the collaboration among doctors, nurses, and pharmacists to streamline the beside dispensing process. The post-intervention phase was conducted for the subsequent 3 months from 1 Apr till 31 Jun 2019.

#### Pre-intervention phase (usual care)

All discharge prescriptions from general medical wards received from 1 Oct till 31 Dec 2018 were retrieved from pharmacy records. Discharge prescriptions received during office hours from 8 am till 5 pm were handled by the pharmacists at the inpatient pharmacy counter, except those that were handled by ward pharmacists will go through the bedside dispensing process.

For each prescription, medication errors with pharmaceutical interventions were recorded. Pharmaceutical interventions encountered in the discharge prescription were divided into three categories, i.e., incomplete prescription, inappropriate/inadequate drug regimen, and miscellaneous. This was based on the categorization and definition in the reporting manual by Pharmaceutical Services Division, Ministry of Health Malaysia [18].

The first category, incomplete prescription, includes missing or incomplete patient’s data, drug’s name, dose, frequency, duration, and doctor’s stamp/or signature in the discharge prescription. Second category, inappropriate/inadequate drug regimen in terms of drug, dose, frequency, duration, and other drug-related problems, e.g., polypharmacy, contraindication, and drug interaction. The miscellaneous category includes drugs given or prescribed to the wrong patient; drugs that are not in the hospital formulary and unclear or illegible handwriting which may lead to wrong interpretation of medication information in the discharge prescription. As ward pharmacists were involved in ward rounds and pharmacotherapy monitoring, prescribing to the wrong patient, i.e., written with the wrong name or identification number could easily be identified through ward registry, and pharmacy computerized system as the record for name and identification number was not tallied. If there were drugs not in the hospital formulary being prescribed, and prescriptions with unclear or illegible handwriting, clarification will be sought immediately from the prescribers.

All identified errors were conveyed to and discussed with the prescribers. The resulting changes or corrections in the discharge prescriptions were done by the prescribers if the interventions were accepted. The medication errors and pharmaceutical interventions recorded by the ward pharmacists were verified by senior pharmacists to ensure validity.

Besides, the number (per tab/vial) of POMs, number of topped-up medication upon discharge, and the costs of medications incurred were recorded to enable calculation of the percentage of cost-saving upon discharge by re-using POMs. The cost of drugs was based on the latest price list obtained from PhIS (Pharmacy Information System). Unit price (MYR) up to 2 decimal points was applied for cost-saving calculation.

#### Intervention phase

The main limiting factor that reduced the rate of bedside dispensing by pharmacists was due to delayed discharge prescription produced by the doctors, in which prescription was not ready during or right after round once discharge was confirmed. The ward pharmacists were unable to capture the discharge prescription as the completion of both discharge summary and the prescription was difficult to predict, which could be in the late afternoon or after office hours. This had resulted in self collecting of discharge medications by patients or caregivers from the hospital pharmacy counter that will be dispensed by the pharmacists after all the discharge documents were completed.

**Fig. 1:**
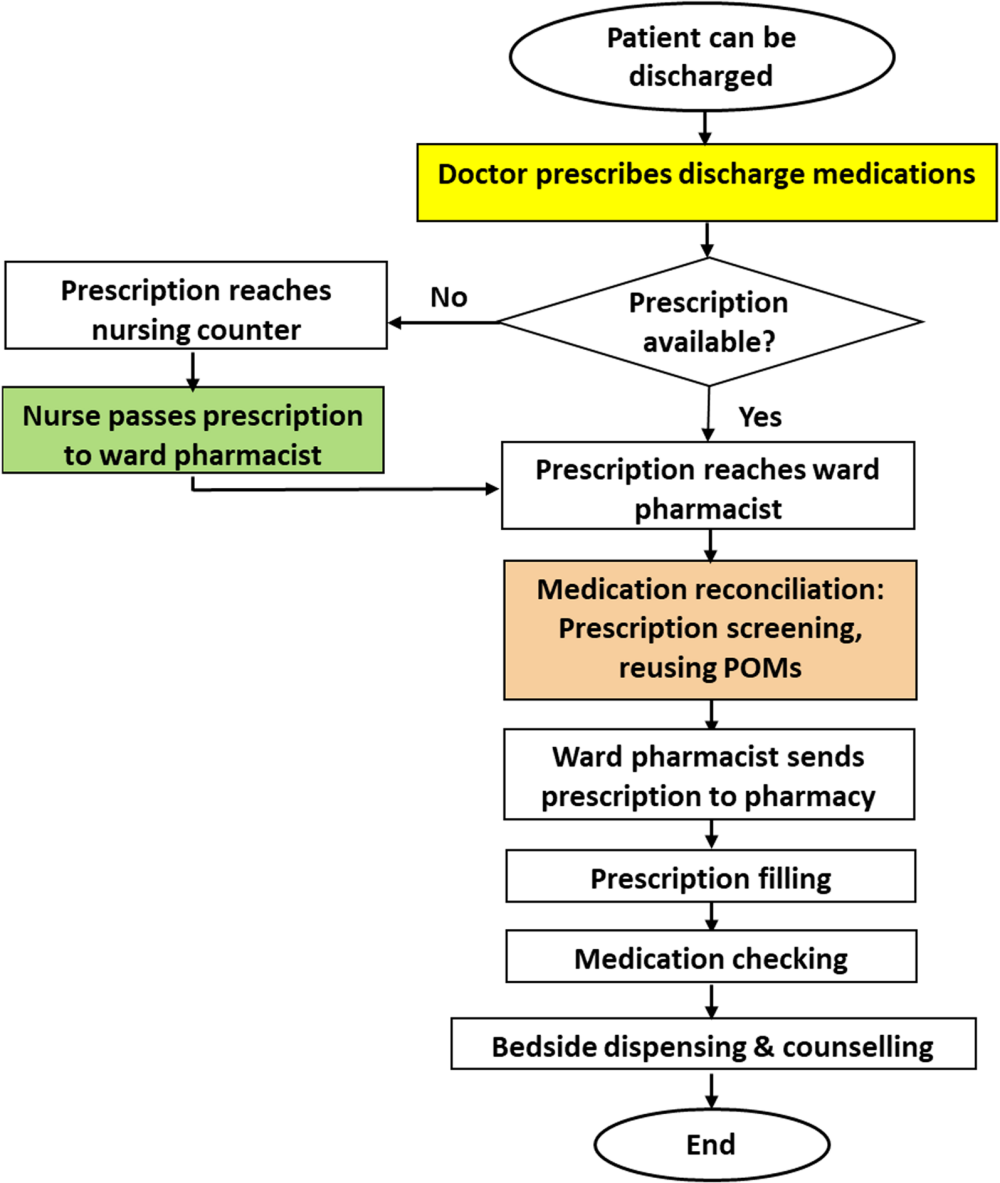
Bedside dispensing process

During the intervention phase, interventional measures were targeted at doctors, nurses, and ward pharmacists who were the key stakeholders involved in the bedside dispensing process, as depicted in Fig. 1.

To streamline the dispensing process, a discussion was held with the head of the medical department/consultants/specialists to strengthen the practice and implementation of planned discharge. Doctors were to prioritize discharge patients and finalize the list of discharge medications with the completion of discharge prescriptions during or right after rounds. For patients that need further review by subspecialties or an undetermined follow-up appointment, discharge prescriptions were to be produced preferably by 3 pm. The prescriptions were then collected and screened by the ward pharmacists or in case there were missed out, the prescriptions were passed to the nurses who handled the discharge service which will then be passed to the ward pharmacists for screening before sending to the inpatient pharmacy for medications preparation. A discussion with the nursing sisters regarding the bedside dispensing workflow had also been conducted before the finalization of the procedure. Once the discharge medications were prepared, bedside dispensing with counseling was provided by the ward pharmacists to the patients in the ward. Pharmacists at the inpatient pharmacy served as backups for bedside dispensing.

A directive letter was ensued to inform all the relevant parties of the implementation of bedside dispensing and counseling services upon discharge following discussions with doctors, nurses, and pharmacists. In the intervention phase, doctors were verbally kept reminded by the ward pharmacists during daily rounds to finalize and produce the discharge prescriptions during or right after the round as far as possible. Nurses were also continuously be reminded regarding the new process.

#### Post-intervention phase

The post-intervention phase was then carried out from 1 Apr till 31 Jun 2019 to assess the effectiveness of the interventional strategy. The same data as in the pre-intervention phase were collected to serve as comparisons.

### Study outcomes

The primary outcome was to determine the rate of bedside dispensing through pharmacist coordinated medication discharge planning on the delivery of BEDISC at post- versus pre-intervention.

Secondary outcomes were to explore medication errors detected and cost savings from using POMs by comparing bedside and counter-dispensing at post- versus pre-intervention. Of note, reusing POMs activity was solely contributed by ward pharmacists.

### Statistical analysis

Statistical analysis was performed using SPSS. Continuous variables were expressed as means (standard deviations) and categorical variables were expressed as numbers and percentages. Descriptive statistics (number and percentage) were used to analyze the data for the number of prescriptions, medication errors, and cost savings from the use of POMs. Data collected with regards to bedside and counter-dispensing in pre- versus post-interventional phases were analyzed using chi-square test and independent samples *t* test where appropriate. All statistical tests were two-tailed and *p* < 0.05 was set to be statistically significant.

## Results

In both pre- and post-intervention phases, more than half of the patients were male with mean age (SD) of 55 (18.5) and 52 (18.8), respectively. As this study was carried out in general medical wards, most of the patients were having multiple comorbidities, such as cardiovascular, endocrine, renal, and respiratory illnesses. The distribution for comorbidities was similar between both phases except those having endocrine (36.9% vs 31.9%) and infection (4.4 vs 2.4%) related illnesses were significantly higher in the pre- versus post-intervention phase. The total number of medications per prescription was around 7, which was similar between the pre- and post-intervention phases (Table 1).

**Table 1:** Characteristics of study populations

Characteristics	Pre-intervention (*n* = 1097)	Post-intervention (*n* = 817)	*p* value
Age, mean (SD)	55 (18.5)	52 (18.8)	0.020*
Gender, *n* (%)			
Male	638 (58.2)	462 (56.5)	0.481
Female	459 (41.8)	355 (43.5)	
Comorbidities, *n* (%)			
Cardiovascular	619 (56.4)	454 (55.6)	0.709
Endocrine	405 (36.9)	261 (31.9)	**0.024**
Renal	396 (36.1)	274 (33.5)	0.245
Respiratory	214 (19.5)	151 (18.5)	0.572
Neurological	80 (7.3)	52 (6.4)	0.428
Rheumatological	78 (7.1)	66 (8.1)	0.427
Infection	48 (4.4)	20 (2.4)	**0.024**
Urological	37 (3.4)	16 (2.0)	0.062
Psychiatric illnesses	29 (2.6)	13 (1.6)	0.120
Malignancy	21 (1.9)	13 (1.6)	0.597
Hematological	10 (0.9)	11 (1.3)	0.366
Gastrointestinal	8 (0.7)	7 (0.9)	0.754
Others	10 (0.9)	9 (1.1)	0.678
Nil	54 (4.9)	63 (7.7)	**0.012**
Number of medications per prescription, mean (SD)	6.8 (3.9)	6.7 (3.9)	0.879*
Dispensing location, *n* (%)			
Bedside	450 (41)	445 (54.5)	** < 0.001**
Counter	647 (59)	372 (45.5)	

Categorical variables were analyzed using chi-square test while continuous variables were analyzed using independent samples *t* test, *p* < 0.05 were considered significant (bold). *p values from independent samples *t* test

**Fig. 2:**
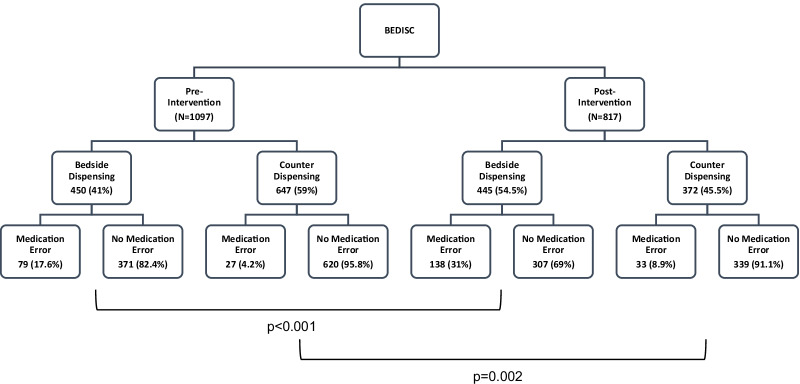
Number of prescription with medication errors detected at bedside and counter dispensing

Throughout the study period, a total of 1097 and 817 discharge prescriptions were dispensed in the pre-intervention and post-intervention phases, respectively. Bedside dispensing rate significantly increased by 13.5% from 41 to 54.5% following remedial actions, consecutively, with reduction of counter-dispensing from 59 to 45.5% (*χ*^2^ = 34.01, d*f* = 1, *p* < 0.001) (Table 1). There was also a significant increase of 13.4% in the detection of medication errors by ward pharmacists at post- versus pre-intervention (31% vs 17.6%) through screening of prescriptions prior to bedside dispensing services (*χ*^2^ = 22.06, d*f* = 1, *p* < 0.001). As for counter-dispensing, a significant increase in medication error detection was shown post-intervention (8.9% vs 4.2%) (*χ*^2^ = 9.41, d*f* = 1, *p* = 0.002). However, the increment of 4.7% was relatively lower as compared to bedside dispensing (Fig. 2).

**Fig. 3:**
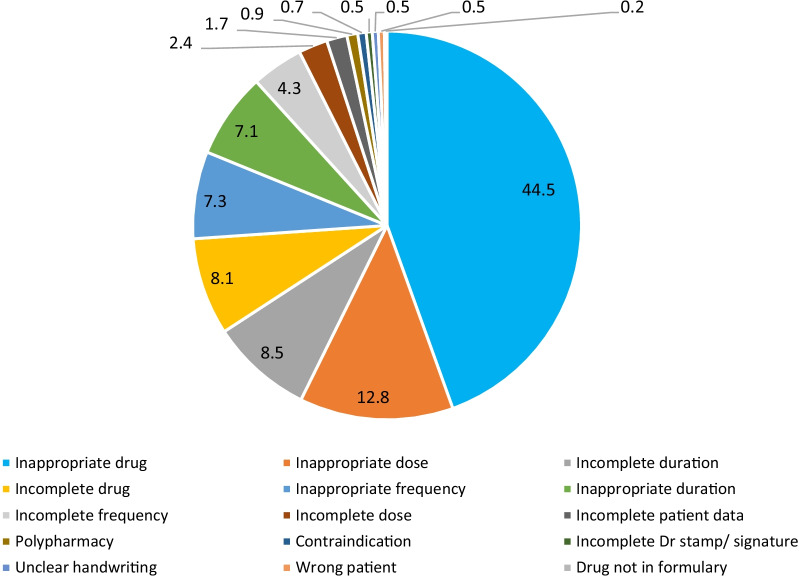
Percentage of medication errors with interventions (*n* = 422)

The number of interventions per prescription for bedside dispensing was higher, 1.6 (129/79 in pre- and 219/138 in post-intervention) in both study phases; compared to 1 (27/27, pre-intervention) and 1.4 (47/33, post-intervention) for counter-dispensing. Overall, the total number of interventions done by ward and counter pharmacists at the pre-intervention phase was 156 and post-intervention was 266. Out of 422 medication errors that led to pharmaceutical interventions, most medication errors fell under the category of inappropriate drug (44.5%, *n* = 188), followed by inappropriate dose (12.8%, *n* = 54) and incomplete duration (8.5%, *n* = 36) (Fig. 3).

**Fig. 4:**
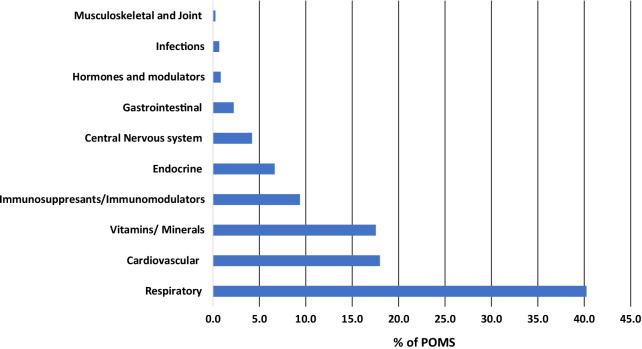
Cost-saving of patient’s own medications (POMs) based on drugs category

Improving BEDISC by reusing POMs resulted in slightly more cost-saving of MYR181.32 at post- versus pre-intervention (MYR6,851.66 at pre-intervention and MYR7,032.98 at post-intervention phase). Reusing POMs saved a total of MYR13,884.64, while topped-up medications contributed to MYR12,482.83 throughout the study period. Therefore, overall cost-saving from reusing POMs in both intervention phases was 52.7%. Despite respiratory illnesses ranked the fourth comorbidity among recruited patients, the majority of cost-saving by reusing POMs was contributed by respiratory medications (40.2%, MYR5,587). This was followed by cardiovascular (18%, MYR2,498.34) and vitamins/minerals (17.5%, MYR2,436.16) (Fig. 4).

## Discussion

Our pre-interventional data revealed a low rate of BEDISC delivery, which principally stemmed from the delayed availability of discharge prescriptions. It is perceived by patients that pharmacy is often responsible for significant delays in hospital discharge. A 2018 UK inpatient survey has revealed that 70% of discharge delays could be attributed to delays in receiving discharge medications and this resulted in increased patient dissatisfaction [19].

We brainstormed the contributing factors for the delayed supply of discharge medications and identified potentially correctable shortcomings in our work process, mainly targeting the doctors who produce the discharge prescriptions and nurses who handle the final discharge paperwork. The post-interventional data have proved that delays in the medication supply process were amenable to early medication discharge planning, which involved a series of interventions led by ward pharmacists to speed up the supply of discharge medications.

To expedite the availability of discharge medications, we revised the existing bedside dispensing process to tackle rate-limiting steps. This would begin with ward pharmacists proactively encouraging doctors to write discharge prescriptions as early as after the decision to discharge was made. In cases where discharge prescriptions were not immediately available, discharge nurses who received them later would directly hand discharge prescriptions to ward pharmacists rather than hand them later to patients together with their discharge summaries. These changes in the work process not only significantly reduced the time for discharge prescriptions to be received by the pharmacy but also allowed ward pharmacists to opportunely perform the on-the-spot review and suggest therapeutic interventions, if necessary, for any drug-related problems in a timely fashion. Consequently, this appeared to shorten the time for discharge medication ready for bedside dispensing. The patient-centered bedside dispensing service is in line with the NHS England Improvement Guide, which underlines the key principles in hastening the supply of discharge medication to improve patient flow and release hospital bed pressures [20].

Our joint effort in streamlining the work procedures and making the availability of discharge prescriptions as early as possible to pharmacy staff had significantly reduced waits and allowed us to achieve a higher rate of bedside dispensing and attendant benefits, including improved medication safety and major cost savings. An Australian hospital has recognized the value of ward pharmacists in ensuring the accuracy of discharge prescriptions. It was observed that ward pharmacists could detect more medication errors as compared to dispensary pharmacists, which was consistent with our findings [21]. Some medication errors could not have been detected by dispensary/counter pharmacists, because ward pharmacists are well-positioned to know patient progress and any medication changes throughout the hospital stay. Ward pharmacists also have access to the information required for medication reconciliation at discharge; for example, inpatient medication charts and pre-admission medication lists.

Most of the medication errors detected upon discharge were under the category of inappropriate/inadequate drug regimen which included either drug omission or wrong drug and dose being prescribed. In our study, we observed an average of 44.5% and 12.8% for drug and dose errors, respectively. The major types of errors detected were consistent with another study done in Malaysia, 34.2% and 28.9%, respectively [22]. Despite the high incidence of medication continuity errors, it had not been linked with an increased risk of rehospitalization [5]. Nonetheless, other outcome such as emergency or urgent care visit was not reported.

It was also interesting to observe the similar category of medication errors detected upon hospital admission and discharge, with relatively higher proportions for drug errors (79.3%) and lower dosing errors (9.6%) during admission in one study conducted in our hospital setting [23]. As we actively perform medication reconciliation through medication history assessment upon hospital admission, the majority of drug errors could have been rectified during admission. Nonetheless, medication reconciliation upon discharge remains important due to changes of drugs or dosing throughout hospital stays, which was proven in this study.

Making use of POMs instead of indiscriminate dispensing for all discharge medications has allowed our center to reduce wastage and save over 50% of drug acquisition costs. The same was observed in a Canadian hospital which practiced POMs, but more cost savings were attained, around 74% [11].

## Limitations

Our study has several limitations despite encouraging findings. We did not explore the impact of BEDISC on patients’ clinical outcomes concerning, e.g., hospital readmission and emergency department visit due to a lack of integrated healthcare information system required to obtain the relevant information.

As successful implementation of BEDISC requires a multidisciplinary team approach, challenges of variable staffing levels, changes in doctors based on clinical rotations, and nurses handling the discharges throughout the study may obscure the effectiveness of the intervention. However, we did ensure a pharmacist backup system was in place and doctors and nurses were continuously reminded about early medication discharge planning and bedside dispensing services to ensure internal validity.

In addition, medication errors detected were case and prescriber-dependent, which may not fully attribute to the effectiveness of the intervention.

Despite these limitations, this study depicted the actual scenario in our normal routine. The pre-post study design is deemed a reasonable option to demonstrate the preliminary evidence for intervention effectiveness, as the initiative driven by pharmacists showed the impact of early medication discharge planning in ensuring the subsequent dispensing and discharge processes can be carried out smoothly with more focuses on medication safety.

## Implications

The significant findings in this study highlighted the importance of the presence of ward pharmacists to provide more holistic patient-centered care. Nonetheless, the determinants of a successful interventional strategy were very much dependent on staffing levels for each stakeholder, awareness of BEDISC, and regular reminders to ensure sustainability. Thus, the benefits gained from these efforts shall be highlighted and communicated to all the stakeholders to gain continuous support and recognition of the importance of BEDISC with early medication discharge planning, and prioritization of discharge handling to improve healthcare service delivery.

## Conclusion

Early medication discharge planning has increased the delivery of bedside dispensing services, consequently enhanced the quality of patient care, patient safety, and reduced medication costs. A multidisciplinary team approach among doctors, nurses, and pharmacists is imperative to ensure early medication discharge planning and bedside dispensing can be carried out efficiently.

## Data Availability

The data sets that support the findings of this study are available from the corresponding author on reasonable request.

## Abbreviations

BEDISC: Bedside dispensing service
MREC: Medical Research and Ethics Committee
PhIS: Pharmacy Information System
POMs: Patient’s own medications
